# A Comparison of Self-Referral and Referral via Primary Care Providers, through Two Similar Digital Mental Health Services in Western Australia

**DOI:** 10.3390/ijerph19020905

**Published:** 2022-01-14

**Authors:** Lauren G. Staples, Nick Webb, Lia Asrianti, Shane Cross, Daniel Rock, Rony Kayrouz, Eyal Karin, Blake F. Dear, Olav Nielssen, Nickolai Titov

**Affiliations:** 1 MindSpot and PORTS Clinics, MQ Health, Faculty of Medicine, Health and Human Sciences, Macquarie University, Sydney 2109, Australia; nick.webb@mq.edu.au (N.W.); lia.asrianti@mq.edu.au (L.A.); shane.cross@mq.edu.au (S.C.); rony.kayrouz@mq.edu.au (R.K.); eyal.karin@mq.edu.au (E.K.); blake.dear@mq.edu.au (B.F.D.); olav.nielssen@mq.edu.au (O.N.); nick.titov@mq.edu.au (N.T.); 2 WA Primary Health Alliance, Psychiatry, Medical School, University of Western Australia, Perth 6907, Australia; daniel.rock@wapha.org.au

**Keywords:** internet, telehealth, anxiety, depression, mental health, primary care, service utilization, implementation

## Abstract

Digital mental health services (DMHSs) deliver mental health information, assessment, and treatment, via the internet, telephone, or other digital channels. The current study compares two DMHSs operating in Western Australia (WA)—The Practitioner Online Referral System (PORTS) and MindSpot. Both provide telephone and online psychological services at no cost to patients or referrers. However, PORTS is accessed by patients via referral from health practitioners, and is designed to reach those who are financially, geographically, or otherwise disadvantaged. In contrast, MindSpot services are available to all Australian residents and patients can self-refer. This observational study compares characteristics and treatment outcomes for patients of PORTS and MindSpot in WA. Eligible patients were people who resided in WA and registered with either clinic from January 2019 to December 2020. Results showed that PORTS patients were more likely to be older, male, and unemployed. They were less likely to report a tertiary education and were more likely to live in areas with higher levels of socioeconomic disadvantage. Despite these differences, treatment outcomes were excellent for patients from both clinics. Results provide further evidence for the accessibility, acceptability, and effectiveness of DMHSs regardless of referral pathway or patient characteristics.

## 1. Introduction

Mental health disorders such as anxiety and depression are significant public health issues worldwide [1,2] and can be exacerbated by barriers to care such as cost, stigma, and limited availability of services [3]. This is particularly true in the current climate, with many traditional health care providers ceasing or reducing face-to-face service delivery because of COVID-19 pandemic restrictions [4,5]. Encouragingly, many randomized controlled trials (RCTs) and meta-analyses have shown that therapist-guided digital mental health services (DMHSs) are effective and acceptable to patients [6,7,8,9,10,11]. Despite the strong evidence base, attempts to integrate DMHSs into primary care systems around the world have met with varying success [12,13], indicating that there remain significant challenges related to service model design, implementation, and regulation. For example, a US-based study observed that the mismatch between traditional and digital health services in the “day-to-day” workflows for staff is a major challenge to successful implementation, requiring specialized and discrete strategies to align traditional and digital services [14]. In a European study extending across six countries (Belgium, France, Germany, Ireland, the Netherlands, and the United Kingdom), gaps in legal and regulatory frameworks were commonly found to limit the wider use of DMHSs [15]. In Sweden, despite national guidelines recommending the use of internet-administered cognitive behavior therapy (CBT) in primary care, less than 21% of surveyed health care centers had implemented it [16]. Similarly in Canada, internet-based CBT is largely unavailable in routine care, despite a brief issued by the Mental Health Commission of Canada in 2014 encouraging the broader use of technology in mental health care [17].

In Australia, the role of primary care in the provision of mental health services is well-recognized [18], as is the utility of integrating DMHSs into primary care frameworks. The Australian Government has funded and encouraged the uptake of DMHSs for several years, and recently committed to increasing funding for the sector [19]. It was one of the first federal governments to adopt an e-Mental Health Strategy [20], and to introduce national safety and quality standards [21]. However, consistent with experiences in other countries, little is known about the factors that ensure the safe, effective, and sustainable integration of DMHSs into the Australian primary health care system [22]. In 2015, the Australian Government recommended a stepped-care model for primary mental health care as a priority area for reform, and Primary Health Networks were established to improve the efficiency and effectiveness of medical services for patients, particularly those at risk of poor health outcomes [23]. In the state of Western Australia (WA), the WA Primary Health Alliance operates the three Western Australian Primary Health Networks (Perth North, Perth South, and Country WA), and in 2017 commissioned the Practitioner Online Referral and Treatment Service (PORTS; www.ports.org.au, accessed on 13 January 2022), a state-wide DMHS for residents of WA aged over 16 years. The PORTS service model was co-designed by the developers of the MindSpot Clinic (www.mindspot.org.au, accessed on 13 January 2022), a national DMHS that provides mental health services to Australian residents aged 18 years and over, funded by the Australian Government.

MindSpot and PORTS provide similar assessment and treatment options, and both are publicly funded and therefore free to patients. However, PORTS is accessed by patients only via direct referral from a primary health care provider and is designed to provide support to people that are traditionally considered hard-to-reach due to financial, geographical, or another disadvantage. In contrast, MindSpot services are not restricted to specific cohorts, and patients do not require a referral to access services. This provides a unique opportunity to test whether DMHSs can successfully target different patient cohorts via different referral pathways [24,25]. Specifically, this paper compares two DMHSs operating in Western Australia between 1 January 2019 to 31 December 2020. The paper aims to (1) provide a descriptive analysis and comparison of patient demographics and characteristics, and (2) compare treatment outcomes for patients who are referred by a health practitioner (PORTS) versus patients who self-refer (MindSpot).

## 2. Materials and Methods

### 2.1. Study Design and Participants

This study was designed as an observational cohort study according to STROBE guidelines [26]. It includes all eligible patients from both clinics who gave consent for their de-identified data to be analyzed for research purposes and who were referred between 1 January 2019 and 31 December 2020. Data from patients accessing MindSpot within this timeframe have been previously used to describe mental health treatment utilization in relation to the COVID-19 pandemic [5,27,28]. Approval to conduct the study was obtained from the Human Research Ethics Committee at Macquarie University (5201200912).

#### 2.1.1. PORTS Patients

People are eligible for PORTS if they are a resident of WA, aged 16 years or older, and are facing disadvantages. Individuals are referred to PORTS by their General Practitioner (GP) or by an approved health practitioner. People are ineligible if they require urgent support, for example, because of imminent suicidal intent or acute psychosis. People requiring immediate or high-intensity support are triaged and on-referred to a more appropriate service, such as an acute crisis service high intensity service.

#### 2.1.2. MindSpot Patients

People seeking assessment or treatment at MindSpot can access the service directly via the website or be referred by a health professional. Approximately 98% of people who register with MindSpot self-refer, although approximately 17% state that they were told about MindSpot by a health professional. MindSpot is a national service that can be accessed by Australian residents from all states and territories, but for the purposes of comparison, only patients who reported a WA postcode were included in the current study. People are eligible for MindSpot if they are 18 years or over, and an Australian resident eligible for publicly funded health services.

### 2.2. Clinical Models

PORTS and MindSpot are funded by the Western Australian Public Health Alliance and the Australian Department of Health respectively, and services are provided free of charge to patients and referrers. Both models were developed in collaboration with consumers, referrers, and researchers, according to the requirements of the funding agencies. Both clinics operate within comprehensive internal and external frameworks that align with Australian National Quality and Health Service Standards [21].

#### 2.2.1. PORTS Clinical Model

In the metro Primary Health Networks (Perth North and Perth South), PORTS accepts GP referrals directly. Metro patients who complete an assessment are contacted by therapists by telephone to discuss assessment and treatment options. This discussion averages 25 min of therapist time and provides an opportunity to discuss a patient’s concerns in greater detail and allows therapists to tailor their advice and recommendations. Metro patients who then select to engage in treatment can choose to enroll in online or telephone-based treatment at PORTS, or they may be referred to a face-to-face psychological service. For patients located in the Country Primary Health Network, which includes the entire state of WA bar metropolitan Perth, PORTS service options are similar, but referral pathways are tailored to the needs of each region of Country WA. For example, PORTS may more directly support mental health services in Country WA during periods of high service demand or health practitioner workforce shortages [25].

PORTS delivers online psychological treatment courses or brief CBT and strengths-based telephone therapy. Both are based on principles of CBT and are delivered by mental health professionals, primarily registered psychologists, who have received training in the delivery of digital mental health services. Other clinical staff are available to therapists for consultation, supervision, and training, including a cultural advisor, an Indigenous advisor, and a psychiatrist.

#### 2.2.2. MindSpot Clinical Model

The MindSpot clinic can be accessed directly via the website (www.mindspot.org.au, accessed on 13 January 2022), which provides information about anxiety and depression, and management of symptoms. People seeking assessment or treatment with MindSpot register by creating an account and completing relevant questionnaires, online or over the phone. People who complete an assessment are invited to discuss their results and treatment options with a therapist. An assessment report that identifies clinically significant symptoms and includes information about how to access mental health or other services, is sent to each patient and, if requested, to a nominated health professional, usually a GP. Patients can also be referred and registered with MindSpot directly by their GP or another health professional. MindSpot therapists are registered mental health professionals, and psychiatrists and cultural consultants are available for consultation.

### 2.3. The Wellbeing Course

The Wellbeing Course is an online transdiagnostic intervention targeting symptoms of both anxiety and depression. The efficacy and acceptability of the course was initially established in a series of RCTs conducted by the eCentreClinic, a Macquarie University online research clinic [7,8,9,11]. Participants in the RCTs showed large clinical improvements in symptoms of depression, generalized anxiety disorder, social anxiety disorder, and panic disorder, with clinical improvements found to be sustained at two-year follow-up. The course is now used as part of routine care at both PORTS and the MindSpot Clinic, and several published reports have demonstrated the effectiveness and acceptability of the course in these real-world settings [5,29,30,31].

In the current study, the Wellbeing Course consisted of five lessons delivered to patients as a series of slides accessible at intervals over eight weeks. The content was designed to teach practical psychological skills and provide relevant psychoeducation.

### 2.4. Questionnaires and Measures

Demographic information was supplied by the referring health professional or the patient at the time of assessment. Postcodes were used to estimate socio-economic disadvantage using the Australian Bureau of Statistics Index of Relative Socio-economic Disadvantage (IRSD) [32]. Patients also answered questions about suicidal ideation, plans, and intent [33]. Standardized and validated self-report questionnaires and semi-structured interviews were used to identify the presence, severity, and duration of symptoms of specific anxiety disorders, depression, and psychological distress, and these were administered at assessment and throughout treatment. For the purposes of this study, treatment outcomes on the Kessler 10-Item Plus Scale (K-10+), the Patient Health Questionnaire 2-Item (PHQ-2), and the Generalized Anxiety Disorder 2-Item Scale (GAD-2) were analyzed. Patients were also asked about their satisfaction with treatment.

The K-10+ was administered at assessment and weekly throughout treatment, including the final week (post-treatment) and three months after treatment (follow-up). The first ten items comprise the K-10 scale, with scores ranging from 10 to 50, with higher scores indicating increasing levels of general psychological distress [34]. The K-10+ also includes four additional items intended to measure the functional impact of reported symptoms. In the current study, the item enquiring about the number of days out of role in the last month was analyzed.

The PHQ-2 is a two-item scale used to assess symptoms of depression [35]. Total scores on the PHQ-2 range from 0 to 6, with scores ≥3 indicative of a depressive disorder [35,36,37]. The GAD-2 is also a two-item scale, with a range from 0 to 6, and a score ≥3 indicating an anxiety disorder [35,36,37,38]. The PHQ-2 and GAD-2 were administered at assessment and throughout treatment, including post-treatment and follow-up, during the Wellbeing Course.

### 2.5. Statistical Analyses

Descriptive analyses and comparison of patient characteristics by clinic were performed. Chi-square analyses of linear-by-linear associations were used to test significance of categorical variables, and ANOVA was used to test significance of continuous variables. Generalized estimating equation models were used to assess the statistical significance of treatment outcomes by clinic and over time. Following intention-to-treat principles and based on previous sensitivity and cross-validation analyses showing that data is likely not missing at random, missing post-treatment and follow-up scores were imputed using separate GEE models with clinic, treatment adherence, and baseline symptoms as covariates [39,40]. An unstructured working correlation matrix and maximum likelihood estimation were used, and gamma distribution with a log link response scale was specified.

Clinical significance was assessed using effect sizes (Cohen’s d) and percentage changes. Negative outcomes were assessed by calculating reliable deterioration at post-treatment on the K-10. Score changes ≥7 were considered reliable [25,41]. A 0.05 significance level was used for all tests, and Bonferroni adjustments were used where required. Deidentified data were analyzed using SPSS version 27 (IBM, Chicago, IL, USA).

## 3. Results

### 3.1. Patient Flow

**Figure 1:**
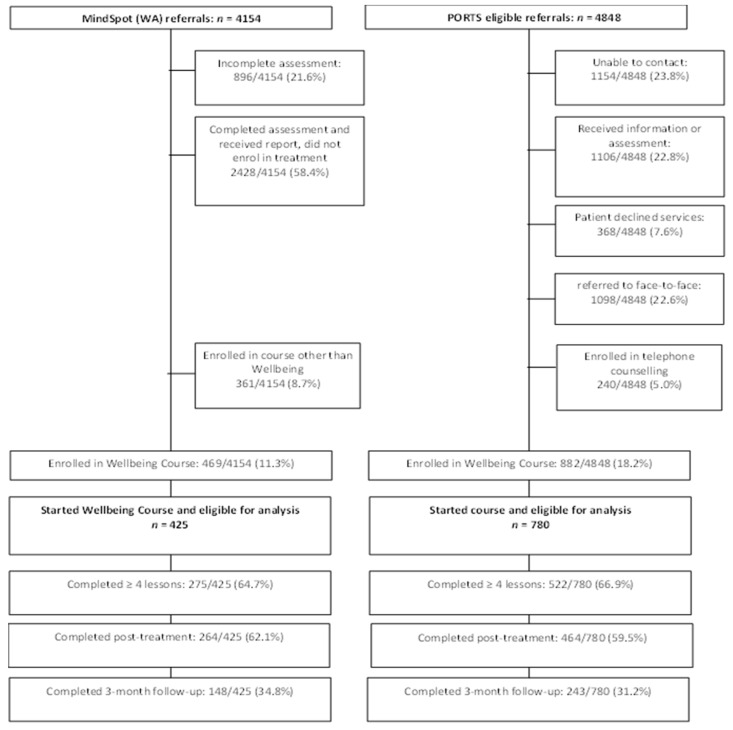
Patient Flow.

Patient flow for both clinics is shown in Figure 1. A total of 5206 referrals were made to PORTS within the study period, of which 4848 (93.1%) consented for analysis. A total of 1655 referrals (34.1%) were from Perth North, 2704 referrals (55.8%) from Perth South, and 489 referrals (10.1%) were from the country. In the same period, a total of 4154 patients from WA started a MindSpot assessment. Most (4080/4154; 98.2%) self-referred, and of these, almost one-third reported that they had never spoken to a health professional about their mental health (1306/4080; 32.0%). Of the 74 MindSpot patients who were directly referred by a health professional, 62 were referred by a GP, 7 by a counsellor, and 5 by a nurse practitioner.

### 3.2. Comparison of Patient Characteristics

There were significant differences between clinics in the demographic characteristics of the patients (Table 1). The mean age of patients from PORTS was significantly higher (39.4 years compared to 34.9 years at MindSpot), and there was a significantly higher proportion of male patients in the PORTS sample (35.5% compared to 24.6%). Patients from PORTS were less likely to have a tertiary education, less likely to be employed, and more likely to be separated, divorced, or widowed. PORTS patients were more likely to reside in urban regions, and in areas of higher socioeconomic disadvantage. Self-reported symptoms of depression and anxiety were higher in the PORTS cohort, but the proportion of patients reporting that they had a current plan for self-harm was significantly lower. Functional interference, indicated by the self-reported number of days patients were unable to undertake their usual activities, was significantly higher for the PORTS cohort (7.7 days out of role per month compared to 5.5 days for the MindSpot cohort).

### 3.3. Wellbeing Course Treatment Outcomes

**Figure 2:**
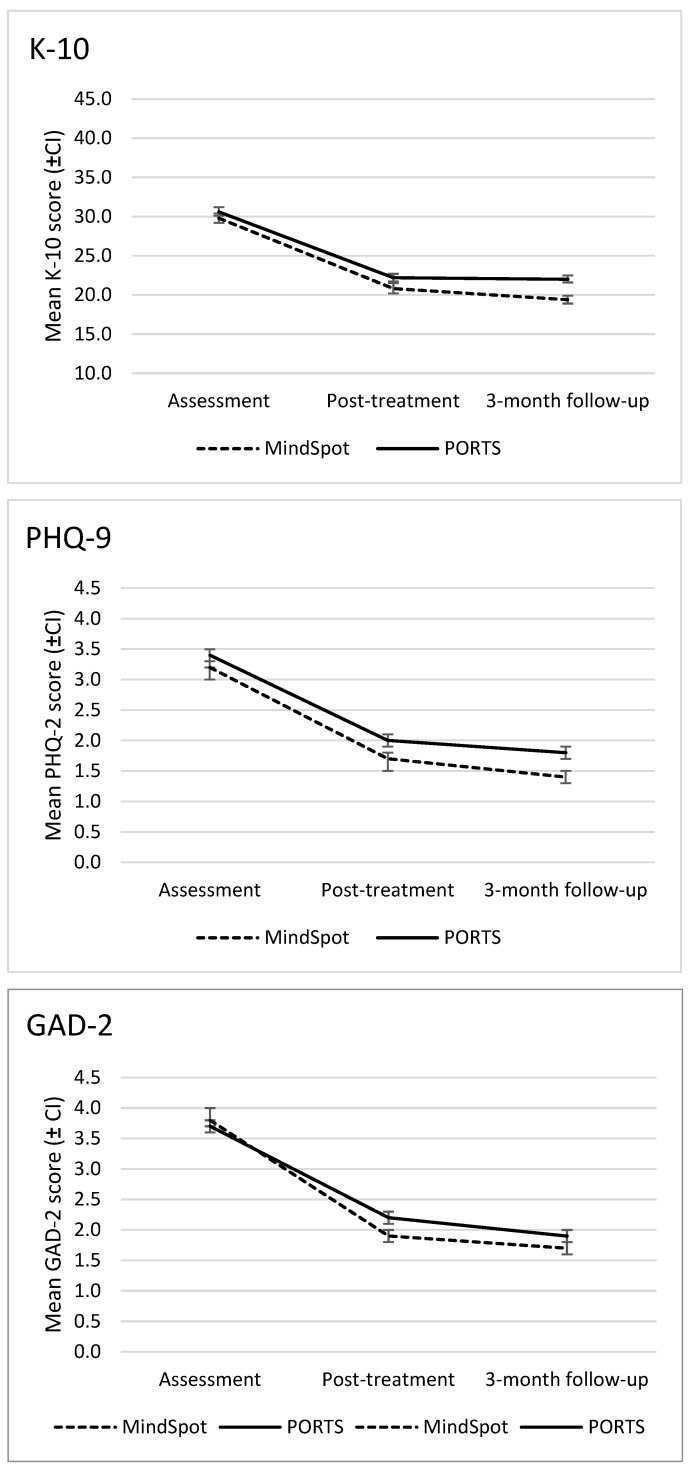
Comparison of symptom reductions for PORTS and MindSpot, from assessment to post-treatment and 3-month follow-up. Error bars represent 95% confidence intervals. Means and standard deviations for each time point are shown in Table 2.

On all symptom measures, patients from both clinics showed significant symptom reductions from assessment to post-treatment and three-month-follow-up on the K10 (Wald’s χ^2^ = 4286.8, *p* < 0.001), PHQ-2 (Wald’s χ^2^ = 1854.5, *p* < 0.001), and GAD-2 (Wald’s χ^2^ = 2379.5, *p* < 0.001). Clinic by time interaction effects were also observed, with the MindSpot cohort showing lower scores than the PORTS cohort at post-treatment and three-month follow-up on all measures (Figure 2).

Estimated means, effect sizes and percentage changes for patients starting the Wellbeing Course are shown in Table 2. Overall effect sizes and percentage changes were large for both clinics. For PORTS, effect sizes ranged from 0.90 to 1.16 at post-treatment, and from 1.03 to 1.28 at three-month follow-up. For MindSpot, effect sizes ranged from 1.00 to 1.34 at post-treatment, and from 1.27 to 1.58 at follow-up. For patients who completed the post-treatment symptom measures, clinical deterioration on the K-10 was low for both clinics: 2.7% (7/264) for MindSpot, and 2.6% (12/463) for PORTS.

Changes in whole days out of role at post-treatment were available for a subset of patients from both clinics (MindSpot: *n* = 107; PORTS: *n* = 263), and these scores were used to identify changes in functional impairment. For MindSpot patients who completed the post-treatment functional interference questions, the mean number of whole days out of role decreased from 6.7 days per month (SD = 7.0) to 3.7 of days per month (SD = 6.8). For PORTS patients, the mean number of whole days out of role decreased from 8.0 days per month (SD = 7.9) to 4.4 days per month (SD = 6.6). Within group effect sizes from assessment to post-treatment were 0.43 (CI 0.16–0.70) for MindSpot and 0.49 (CI 0.32–0.67) for PORTS.

### 3.4. Treatment Satisfaction

A total of 691 patients completed the treatment satisfaction questionnaires (259 from MindSpot and 432 from PORTS). Of the MindSpot patients, 95.8% (248) would recommend the Wellbeing Course to a friend, and 96.1% (249) reported that it was worth their time. Of the PORTS patients, 93.8% (405) would recommend the course to a friend, and 94.7% (409) stated that it was worth their time.

## 4. Discussion

PORTS and MindSpot are two DMHSs providing telephone and online psychological assessments, treatment and referral services to patients located in WA, Australia. Both clinics utilize comparable assessment, treatment, and data management processes, but have different referral pathways. PORTS is accessed by patients through referral from a primary health care provider and is designed to reach those who are financially, geographically, or otherwise disadvantaged. In contrast, MindSpot services are not targeted to a specific cohort and do not require referral. The current study compared patient characteristics and treatment outcomes for both clinics. Results showed that the clinics serve patients with significantly different demographic, socioeconomic, and geographic features. Despite these differences, outcomes were excellent for patients accessing online treatment through either clinic.

PORTS patients were more likely to be older, male, unemployed, and less likely to report a tertiary education. They reported more severe symptoms of anxiety and depression, and geographic indicators suggested higher levels of socioeconomic disadvantage. The pattern of observed patient characteristics was unsurprising given that PORTS is targeted toward people facing socioeconomic or geographic disadvantage or limited access to services for other reasons, especially those in remote locations. A pleasing feature of the PORTS cohort was the greater number of male patients, who are under-represented in outpatient mental health care settings. In contrast, MindSpot patient demographics corresponded closely with those of a recent survey of primary care consumers in Australia, which found that people using mental health websites were more likely to be female, younger, highly educated, and employed [42].

A higher proportion of MindSpot patients reported a current plan for self-harm (3.3% of MindSpot respondents compared to 1.9% of PORTS respondents). This may be expected because of the differences in referral pathways. Given that MindSpot patients usually self-refer and may never have previously spoken with a health professional, there is no opportunity to screen for suicidal thoughts or intentions until the assessment questions are answered. In contrast, individuals are referred to PORTS by their GP or health practitioner. This means they are more likely to have received a risk assessment by their primary health care provider and been referred to a more appropriate service if at imminent risk. Encouragingly, it should be noted that both PORTS and MindSpot have detailed risk assessment procedures in place for any patients who do disclose suicidal plans or intent [33].

A key finding from the current study was that both DMHSs were effective in providing triage, support, and education, as well as treatment. Many patients reported mainly seeking assessment and information about mental health conditions, which served as a brief intervention. For both clinics, around one in five patients enrolled in an online treatment course. Previous research indicates that many patients with symptoms of anxiety or depression do not initiate mental health treatment despite treatment availability [24,25,43]. This is likely due to complex interactions between attitudinal and structural barriers, and patient demographics and preferences [44]. The factors associated with treatment uptake for patients of MindSpot is a focus of current research [45], and this remains an important area for further investigation.

For patients who did enroll in the online Wellbeing Course through either clinic, treatment outcomes were excellent. Patients accessing online treatment via PORTS generally had more severe symptoms than those accessing the course via MindSpot, however effect sizes were large for both clinics, and symptoms reduced by 40% to 56%. Clinical deterioration on the K-10 was less than 3% for both clinics, and patient satisfaction was high. These outcomes are consistent with previous reports [24,25], and compare favorably with outcomes from other DMHSs that offer online treatment as part of routine care in public health systems around the world [46,47,48,49,50,51].

An important feature of DMHSs is the systematic collection of data, used for benchmarking and quality assurance, and to inform policy and funding decisions. The main strength of this study is the availability of comprehensive, real-world data, from two concurrently operating DMHSs using the same methods of data collection and analyses, and offering similar services, but via different health care pathways. This provided a unique opportunity to directly compare clinical flow, patient characteristics, and treatment outcomes within two publicly funded DMHSs, one of which targeted a distinct cohort of patients.

There are some limitations to the study. Interpretation of the results is limited by missing data, due to patients who were unable to be contacted, declined permission to analyze their data, or did not complete post-treatment or follow-up questionnaires. Missing data is a limitation of many studies, particularly those involving people who are accessing a service rather than participating in a research trial. However, the weekly collection of symptom scores during treatment and appropriate statistical modelling attempted to mitigate this limitation. It is also important to note that MindSpot and PORTS are well-established and well-regulated DMHSs, and services are delivered by trained mental health professionals, which may limit the generalizability of the results. Similarly, this study was restricted to a sample of patients from WA and different patterns may be found in other jurisdictions. Future research and evaluations are required to replicate these results in other primary health care settings, to ensure that evidence-based care is routinely available to patients in efficient and cost-effective ways.

## 5. Conclusions

Results showed that PORTS was able to successfully engage otherwise hard to reach populations via primary care referral pathways. Further, treatment outcomes were comparable to the MindSpot Clinic, where patients can access services directly. The implementation of DMHSs in routine care is a considerable challenge and requires clear evidence to assist those involved in service planning and policy development. It is hoped that the consistency of outcomes reported here, achieved via two different service models, will help Governments in Australia and other jurisdictions when developing their own models. Results provide further evidence for the accessibility, acceptability, and effectiveness of DMHSs regardless of referral pathway or patient characteristics.

## Figures and Tables

**Table 1:** Demographic characteristics of patients referred to MindSpot (*n* = 4154) and PORTS (*n* = 4848).

	MindSpot	PORTS	Sig.
Age			
Mean age (SD)	34.9 (13.6)	39.4 (15.8)	F = 211.3; *p* < 0.001 ***
Age range	18–93 years	16–95 years	
Gender			
Female	74.7% (3103/4154) ^a^	62.6% (3034/4848) ^b^	χ^2^ = 158.9, *p* < 0.001 ***
Male	24.6% (1021/4154) ^a^	35.5% (1722/4848) ^b^	
Other	0.7% (30/4154) ^a^	1.9% (92/4848) ^b^	
Cultural identity			
Aboriginal or Torres Strait Islander	3.1% (118/3832)	3.6% (95/2644)	χ^2^ = 3.2, *p* = 0.199
Born in Australia: non-Indigenous	67.8% (2589/3832)	65.8% (1741/2644)	
Born in country other than Australia	29.1% (1116/3832)	30.6% (808/2644)	
Education			
Postgraduate degree	15.6% (602/3871) ^a^	5.9% (156/2644) ^b^	χ^2^ = 321.8, *p* < 0.001 ***
Undergraduate degree	21.6% (837/3871) ^a^	11.7% (310/2644) ^b^	
Other tertiary qualification	29.9% (1157/3871) ^a^	33.4% (883/2644) ^b^	
No tertiary qualification	32.9% (1275/3871) ^a^	49.0% (1295/2644) ^b^	
Employment			
Employed full or part-time	57.4% (2226/3877) ^a^	42.2% (1116/2644) ^b^	χ^2^ = 145.6, *p* < 0.001 ***
Unemployed	12.0% (465/3877) ^a^	16.0% (423/2644) ^b^	
Other	30.6% (1186/3877) ^a^	41.8% (1105/2644) ^b^	
Marital status			
Married (registered or de facto)	37.5% (1453/3871) ^a^	34.1% (902/2644) ^b^	χ^2^ = 90.9, *p* < 0.001 ***
Separated	5.8% (225/3871) ^a^	10.1% (266/2644) ^b^	
Divorced	5.8% (225/3871) ^a^	7.6% (201/2644) ^b^	
Widowed	0.7% (28/3871) ^a^	2.5% (66/2644) ^b^	
Other	50.1% (1940/3871) ^a^	45.7% (1209/2644) ^a^	
Self-described location			
Capital city or surrounding suburbs	71.3% (2757/3869) ^a^	74.8% (1977/2644) ^b^	χ^2^ = 37.8, *p* < 0.001 ***
Other urban area	11.2% (435/3869) ^a^	13.2% (348/2644) ^b^	
Rural or remote region	17.5% (677/3869) ^a^	12.1% (319/2644) ^b^	
IRSD			
First quintile (most disadvantaged)	10.0% (402/4031) ^a^	16.1% (777/4826) ^b^	χ^2^ = 173.2, *p* < 0.001 ***
Second quintile	23.0% (926/4031) ^a^	26.6% (1286/4826) ^b^	
Third quintile	20.2% (814/4031) ^a^	23.0% (1109/4826) ^b^	
Fourth quintile	20.9% (844/4031) ^a^	16.5% (798/4826) ^b^	
Fifth quintile (least disadvantaged)	25.9% (1045/4031) ^a^	17.7% (856/4826) ^b^	
Symptoms and impairment			
General distress (K-10)	31.1 (7.4)	31.5 (8.0)	F = 2.8, *p* = 0.093
Self-reported current depression	69.7% (2662/3820)	78.3% (2071/2645)	χ^2^ = 0.59.1, *p* < 0.001 ***
Self-reported current anxiety	87.3% (3333/3820)	89.7% (2373/2645)	χ^2^ = 0.9.2, *p* = 0.002 **
Thoughts of suicide in last week	29.1% (1051/3615)	27.2% (720/2644)	χ^2^ = 2.6, *p* = 0.110
Current plan for self-harm	3.3% (118/3615)	1.9% (50/2644)	χ^2^ = 11.0, *p* < 0.001 ***
Whole days out of role in last month	5.5 (7.5)	7.7 (8.9)	F = 94.1, *p* < 0.001 ***

** Significant at *p* < 0.01; *** Significant at *p* < 0.001. Each superscript ^(a,b)^ letter denotes a subset whose proportions differ significantly from other subsets at the 0.05 level.

**Table 2:** Treatment outcomes for patients enrolled in the Wellbeing Course at MindSpot (*n* = 425) and PORTS (*n* = 780).

		Estimated Marginal Means			Effect Size from Assessment		Percentage Change from Assessment	
	*n*	Assessment	Post-Treatment	3-Month Follow-Up	To Post-Treatment	To 3-Month Follow-Up	To Post-Treatment	To 3-Month Follow-Up
K-10								
MindSpot	425	29.8 (6.7)	20.8 (6.9) ***	19.5 (5.4) ***	1.32 [1.17–1.47]	1.69 [1.53–1.85]	45.2% [41.8%–48.4%]	52.4% [49.8%–55.0%]
PORTS	780	30.6 (7.4)	22.2 (7.5) ***	22.0 (6.4) ***	1.13 [1.02–1.23]	1.24 [1.13–1.35]	40.9% [38.3%–43.5%]	41.8% [39.6%–44.0%]
PHQ-2								
MindSpot	425	3.2 (1.6)	1.7 (1.4) ***	1.4 (1.2) ***	1.00 [0.85–1.14]	1.27 [1.12–1.42]	47.9% [43.6%–52.0%]	56.1% [52.5%–59.4%]
PORTS	780	3.4 (1.8)	2.0 (1.5) ***	1.8 (1.5) ***	0.85 [0.74–0.95]	0.97 [0.86–1.07]	41.4% [38.2%–44.5%]	46.5% [43.3%–49.4%]
GAD-2								
MindSpot	425	3.8 (1.6)	1.9 (1.4) ***	1.7 (1.1) ***	1.26 [1.12–1.41]	1.53 [1.38–1.68]	50.1% [46.6%–53.5%]	54.6% [51.6%–57.3%]
PORTS	780	3.7 (1.7)	2.2 (1.5) ***	1.9 (1.2) ***	0.94 [0.83–1.04]	1.22 [1.11–1.33]	40.5% [37.6%–43.3%]	48.8% [46.5%–51.1%]

Standard deviations are shown in round parentheses for the estimated marginal means, and 95% confidence intervals are shown in square parentheses for the effect sizes and percentage changes. *** Significant at *p* < 0.001 compared assessment.

## Data Availability

Access to de-identified data may be provided to external researchers upon reasonable request. Requests must be made in writing and are subject to the establishment of appropriate data governance, and the approval of an independent and recognized Human Research Ethics Committee.
